# Safety Assessment of Rehabilitation Robots: A Review Identifying Safety Skills and Current Knowledge Gaps

**DOI:** 10.3389/frobt.2021.602878

**Published:** 2021-03-22

**Authors:** Jule Bessler, Gerdienke B. Prange-Lasonder, Leendert Schaake, José F. Saenz, Catherine Bidard, Irene Fassi, Marcello Valori, Aske Bach Lassen, Jaap H. Buurke

**Affiliations:** ^1^ Roessingh Research and Development, Enschede, Netherlands; ^2^ Department of Biomedical Signals and Systems, University of Twente, Enschede, Netherlands; ^3^ Department of Biomechanical Engineering, University of Twente, Enschede, Netherlands; ^4^ Fraunhofer Institute for Factory Operation and Automation, Magdeburg, Germany; ^5^ Interactive Robotics Laboratory, CEA LIST, Palaiseau, France; ^6^ National Research Council of Italy, Milan, Italy; ^7^ Department of Robot Technology, Danish Technological Institute, Odense, Denmark

**Keywords:** rehabilitation robots, physical human-robot interaction, safety assessment, hazards, regulation, standardization, mechanical safety, development phase

## Abstract

The assessment of rehabilitation robot safety is a vital aspect of the development process, which is often experienced as difficult. There are gaps in best practices and knowledge to ensure safe usage of rehabilitation robots. Currently, safety is commonly assessed by monitoring adverse events occurrence. The aim of this article is to explore how safety of rehabilitation robots can be assessed early in the development phase, before they are used with patients. We are suggesting a uniform approach for safety validation of robots closely interacting with humans, based on safety skills and validation protocols. Safety skills are an abstract representation of the ability of a robot to reduce a specific risk or deal with a specific hazard. They can be implemented in various ways, depending on the application requirements, which enables the use of a single safety skill across a wide range of applications and domains. Safety validation protocols have been developed that correspond to these skills and consider domain-specific conditions. This gives robot users and developers concise testing procedures to prove the mechanical safety of their robotic system, even when the applications are in domains with a lack of standards and best practices such as the healthcare domain. Based on knowledge about adverse events occurring in rehabilitation robot use, we identified multi-directional excessive forces on the soft tissue level and musculoskeletal level as most relevant hazards for rehabilitation robots and related them to four safety skills, providing a concrete starting point for safety assessment of rehabilitation robots. We further identified a number of gaps which need to be addressed in the future to pave the way for more comprehensive guidelines for rehabilitation robot safety assessments. Predominantly, besides new developments of safety by design features, there is a strong need for reliable measurement methods as well as acceptable limit values for human-robot interaction forces both on skin and joint level.

## Introduction

Rehabilitation robots have become increasingly relevant in the past years as new technologies are becoming available and with an increasing need for physical rehabilitation. With the world population aging and chronic disabilities becoming more frequent as a consequence (World Health Organization, 2018a; World Health Organization, 2018b), a lack of skilled clinicians is expected to develop.

In this article, we are using the term rehabilitation robotics as an overarching term that refers to a “medical robot intended by its manufacturer to perform rehabilitation, assessment, compensation or alleviation comprising an actuated applied part” (International Organization for Standardization, 2019b). The actuated applied part is an important feature of a rehabilitation robot. It means that there is a part of the robot which is in contact with the human and intended to provide physical interaction, driven by an actuation system and controlled by the robot alone or in a shared control of robot and patient. There is a wide range of device types that fall under this description and that can be classified in various ways. One way to classify them is by their intended use (i.e., rehabilitation, assessment or assistive), which however can be ambiguous as many devices can be used both in a rehabilitation or training situation and in a home environment as an assistive device (Radder et al., 2019). They can be further classified according to their mobility (i.e., fixed or stationary devices vs. mobile/ambulatory/wearable devices), the targeted body part (upper limb vs. lower limb) and the mechanical setup of each device (e.g., exoskeletons, end-effectors, soft exosuits). Due to considerable research efforts in the field, there are constantly new devices developed and new device types evolving. Therefore, one might need to refine and/or extend the classification of rehabilitation robots in the future (Shirota et al., 2017). Nonetheless, the most common device types will be introduced in the following sections.

### Types of Rehabilitation Robots

Exoskeletons are rigid anthropomorphic structures that are attached to a human’s body segments by the means of cuffs or straps. The exoskeleton’s rigid segments are usually attached to the lateral sides of the patient’s limbs. The actuation is often either achieved through servo or DC motors at the joints or through cable driven systems (Gull et al., 2020). They can be used for different body parts including hand, arms and legs and can be either part of a stationary system, e.g. ArmeoPower for upper extremity and Lokomat for lower extremity (both Hocoma, Volketswil, Switzerland) or wearable and mobile, e.g., MyoPro arm exoskeleton (Myomp, Cambridge, MA, United States) and ReWalk lower limb exoskeleton (ReWalk Bionics, Marlborough, MA, United States).

Exosuits are soft robots that act in a similar way as exoskeletons. However, instead of being built out of rigid structures, they are largely made from soft materials like fabric. Common actuation systems include variable stiffness actuators, series elastic actuators and pneumatic actuators (Sanchez-Villamañan et al., 2019; Gull et al., 2020). Exosuits can be used for the upper limb, often as a glove like the Carbonhand (Bioservo Technologies, Kista, Sweden) or for the lower limb like the Myosuit (MyoSwiss, Zurich, Switzerland).

Rehabilitation robot systems based on an end-effector are usually attached to a distal segment of the patient and can, similarly to exoskeletons, be used for upper or lower limbs. While this rehabilitation robot type to some extent is comparable with (collaborative) robot arms in the industrial domain, the shapes and forms of end-effector-type devices in healthcare can be much more diverse. There are devices which make use of an industrial robot arm as the basis. Robot arm type devices for upper limb rehabilitation can for example be used for assessing or training the range of motion of a patient sitting in a chair, such as Burt (Barrett Technology, Newton, MA, United States), and those for lower limbs can be used for mobilization of patients’ legs, such as ROBERT (Life Science Robotics, Aalborg, Denmark). There are other end-effector-type devices which do not have the shape of a robot arm and are often based on a haptic interface, such as the InMotion ARM (Bionik Laboratories, Toronto, Canada). End-effector based gait trainers such as the G-EO (Reha Technology, Olten, Switzerland), are usually used in combination with a body-weight support system.

Robots for body-weight support are oftentimes used for gait rehabilitation. They can be used as an independent system which is connected to the ceiling via a railing system such as the ZeroG (Aretech, Ashburg, VA, United States) or FLOAT (Reha-Stim Medtec, Schlieren, Switzerland), in combination with a mobile robot (see below) or as part of a stationary gait trainer (exoskeleton or end-effector). However, body-weight support systems which are built-in subsystems of a stationary or mobile gait trainer robot are not necessarily robots. They can also be dynamic weight lifters without any sensing function or autonomy. There are also robotic arm support systems like the ExoArm (Focal Meditech, Tilburg, Netherlands), which provide an active weight support for the patient’s arm and can for example be attached to a wheelchair.

Mobile platform robots are used for gait rehabilitation. They can be combined with a body-weight support to bridge the gap between stationary gait trainers and overground gait training like the Andago (Hocoma, Volketswil, Switzerland). Another type of mobile rehabilitation robot is a robotic walker or cane.

Balance trainers are robots that can be used for balance training and often include a platform (fixed, mobile or in the form of a treadmill) and a weight support system which are programmed to disturb the patient’s balance. Examples are the Balance Training Assist (Toyota Motor Corporation, Toyota, Japan) and the Balance Tutor (MediTouch, Netanya, Israel).

### Rehabilitation Robots as Collaborative Robots

By definition, a collaborative robot is a robot that works in close interaction with a human. Oftentimes, the task of the collaborative robot is to take over the heavy lifting or repetitive tasks from the workers. Therefore, a rehabilitation robot, which takes over physically demanding or repetitive tasks from the therapist, can be seen as one type of collaborative robot. However, while collaborative robots in industry work together with factory workers, robots in rehabilitation have two very different main types of users: patients and therapists. The therapist can be seen as the equivalent to the factory worker in this comparison as the rehabilitation robot is performing tasks like supporting the patient in repetitive movements or supporting the patient’s body weight. The patient has a different role as he or she is usually physically attached to the robot in contrast to the therapist who is standing in close proximity or at most touching the robot with the hands. Safety is an inherent challenge of rehabilitation robots, which does not only include the occupational safety of the therapist but also the safety of the patient who is strapped to the powerful machine that is the robot. In addition to the close interaction, the individual characteristics of each patient are an issue. Pathologies can change pain perception or cause sudden or prolonged movement restrictions, all of which are aspects that can introduce risks. Moreover, rehabilitation robots like wearable exoskeletons can be used in an uncontrolled environment, as for example a patient’s home or a park, which introduces additional hazards that are not present in a controlled environment such as a factory floor.

### Implementation Barriers

There has been a lot of development of new technologies for rehabilitation robotics in the recent years. However, a number of implementation barriers remain. In addition to ongoing developments in the fields of actuation and mechanical design (Calabrò et al., 2016; Sposito et al., 2019), there are still limited solutions available for recognizing the user’s intent for movement and using it as a control input (Herr, 2009; Calabrò et al., 2016). Moreover, rehabilitation robots are not able to perfectly mimic the movements of the human body. Joints of exoskeletons are often simplified approaches to mimic the movement of the human joints which makes the exoskeleton over-constrained and leads to limitations in degrees of freedom. End-effector-type devices on the other hand are under-constrained which is why they might not offer enough support for more severely affected patients (Calabrò et al., 2016).

Beyond those practical barriers, there are some barriers regarding safety. To achieve a comfortable and effective interaction between robot and patient, the mechanical interface needs to be designed in a way that it is compliant enough to ensure comfort and avoid injuries and at the same time stiff enough to transfer the forces to the patient’s musculoskeletal system to achieve the intended effect of the robot (Herr, 2009; Calabrò et al., 2016). Moreover, the mismatch between robot and human joints as addressed above is also an aspect that needs to be considered when discussing safety. A misalignment between the joints of the patient and the robot can lead to unwanted interaction forces, which can potentially be unsafe (Rocon et al., 2008). Another type of misalignment is unavoidable and related to the oversimplification of the exoskeleton joints. While the flexion axis of the human knee joint for example is displaced during knee flexion, an exoskeleton’s knee joint is typically realized by a simple hinge. Therefore, there is a misalignment building during each step (Akiyama et al., 2012). End-effector-type rehabilitation robots offer more degrees of freedom and are under-constrained. Therefore, misalignment as it occurs in exoskeleton devices is avoided in end-effector-type devices. However, due to the under-constrained nature of end-effector-type devices, they can impose unnatural movements on the wearer which might also lead to excessive forces on the musculoskeletal system (Rocon et al., 2008). Recent studies investigating potential effects of interaction between humans and robots (Behrens and Elkmann, 2014; Mao et al., 2017b), do not focus on rehabilitation robots. Interaction with rehabilitation robots, as opposed to other collaborative robots, is characterized by prolonged physical contact with cyclic loading and unloading and vulnerable users. This underlines the need for carefully considering potential negative effects of the human-robot interaction in the rehabilitation domain and investigating how to avoid or minimize them.

Before rehabilitation robots can be made commercially available in the European Union (EU), they need CE certification. To achieve that, the manufacturer needs to demonstrate that their device is safe. However, safety validation of rehabilitation robots is complex. This is partly due to the fact that the field of rehabilitation robots is a rather new field, which reduces the availability of best practices and applicable safety standards. Especially when it comes to explicit testing procedures that can be used during robot development, information in regulations and standards is rare, or scattered across multiple standards. The familiarization with applicable regulations and standards and the process of safety validation takes a lot of time, which can be a burden, especially for small to medium enterprises and start-ups.

The assessment of rehabilitation robot safety is a vital aspect of the development process, which is often experienced as difficult. Safety of rehabilitation robot use in clinical trials, including the monitoring and reporting of adverse events (Mehrholz et al., 2017; Mehrholz et al., 2018), is one important aspect. However, this has been covered elsewhere (Bessler et al., 2020) and will therefore not be addressed in this article. This article is instead focusing on guidance for validating mechanical safety of rehabilitation robots in the development phase, as required for the risk management process (International Organization for Standardization, 2010; International Organization for Standardization, 2019a).

To provide directions for developers as a guideline to evaluate rehabilitation robot safety, this article first gives an overview of the state of the art in rehabilitation robot safety validation. Key information on the regulatory background and safety certification process is summarized and a new concept for a cross-domain knowledge platform on safety validation is introduced. Then, we will identify the most relevant hazards when using rehabilitation robots based on a recent systematic literature review and additional literature. Next, we explain how those hazards are translated to so-called safety skills. These are abstract representations of safe target behaviors of a robot that can be validated by executing structured testing protocols. This article concludes by identifying the most pressing gaps and needs that have to be overcome and recommending ways to achieve this.

## Rehabilitation Robot Safety Validation – State of the Art

### Regulatory Background

In the EU the legislation for medical devices applies for rehabilitation robots. In 2017, EU regulation 2017/745 (European Parliament and Council of the European Union, 2017), also known as the Medical Device Regulation (MDR), was accepted by the European Parliament. The MDR is replacing council directives 90/385/EEC and 93/42/EEC, also known respectively as the Active Implantable Medical Devices Directive (AIMD) and the Medical Device Directive (MDD), where the MDD was the relevant legislation for rehabilitation robots. On the May 26, 2017, a transition period started to enable companies, developers and Notified Bodies to take the appropriate measures to comply with the MDR, which will become fully operational on the May 26, 2021 (European Parliament and Council of the European Union, 2020).

Where the MDD appeared to focus mainly on safety of a medical device during the design process (Mishra, 2017), the MDR emphasizes safety and performance during its entire lifetime. This is apparent in a number of articles focusing on post-market surveillance, including required periodic post-market safety reports. The level of detail needed for this post-market evaluation depends on the risk classification of the medical device and the methodology has to be adequately defined by the manufacturer before the device can receive CE marking. So, where for a class I device a post-market surveillance strategy consisting of user surveys could suffice, class III devices could also require a more elaborate post-market strategy that involves the collection of data related to performance and safety of the device in use. The MDR also adds a strong focus on the performance of the medical device, being more prescriptive than the MDD on how medical claims of the device can be proven, how to conduct clinical investigation in the pre-market phase, and extending it to post-market surveillance as well (e.g., post market clinical follow-up, PMCF). The goal for this is that all claims with respect to clinical performance as stated by the manufacturer have to be supported by clinical data but also to evaluate and improve the initial risk analysis, thus providing additional information for the risk/benefit analysis. These processes should be properly described for the risk management process, as described in ISO 14971 *Medical devices—Application of risk management to medical devices* (International Organization for Standardization, 2019a). The clinical data to support the clinical performance claims can be collected during clinical studies. In essence, the requirements in the MDR for conducting clinical studies follow the Good Clinical Practice (GCP) guidelines for medical devices (International Organization for Standardization, 2020a). The MDR also contains a clear definition for clinical data to support the clinical performance claims, which is also more prescriptive than in the MDD.

As an aid for demonstrating conformity, standards are often used. For active medical devices, usually the standard IEC 60601–1 *Medical electrical equipment - Part 1: General requirements for basic safety and essential performance* is the standard to use, including the additional standards from the IEC 60601–1 series, like the EN-IEC 60601-1-2, IEC 60601-1-10, etc. Over the past years many different domain-specific standards (IEC 60601-2-xx and IEC 80601-2-xx series) have been developed in addition to IEC 60601-1, which translate the general safety and performance requirements from the IEC 60601-1 into more domain specific safety and performance requirements. In 2019 a new domain-specific standard has been published for the rehabilitation robots domain. This standard (IEC 80601-2-78 *Medical electrical equipment—Part 2–78: Particular requirements for basic safety and essential performance of medical robots for rehabilitation, assessment, compensation or alleviation*) is a domain-specific standard that clarifies a number of items specific to rehabilitation robots, that are not clearly addressed in the IEC 60601-1 or for which interpretation of the IEC 60601-1 can be complicated, e.g., for active applied parts, the definition of support systems etc.

When a device complies with relevant so called harmonized standards, the developer can assume that the device is in agreement with the EU legislation. However, for medical devices the current relevant harmonized standards are harmonized for the MDD. At the time of writing this paper, no standards that have been harmonized under the MDR have been published yet in the Official Journal of the European Union. The EU has published a timeline for harmonization of standards according to the MDR. The harmonization deadline for process related standards (e.g. ISO 13485, ISO 14971, ISO 14155) and for labeling requirements (ISO 15223-1 and EN 15986) is the May 26, 2020, while the timeline for harmonization of more technical standards ranges between September 2021 and May 2024 (European Commission, 2019). This means that for the period between May 2021 and May 2024 there probably will be no or just a limited number of harmonized standards that can officially be used to demonstrate conformity with the MDR. Manufacturers therefore should discuss with their notified body at an early stage the methods to demonstrate conformity with the MDR to avoid additional costs during the CE process.

Manufacturers of rehabilitation robots should also be aware that article 1.6 of the MDR in essence states that devices that can also be seen as machinery (such as a robot) should also meet essential health and safety requirements as set out in Annex I of the Machinery Directive (European Parliament and Council of the European Union, 2017). This is particularly relevant for demonstrating conformity for CE, since some of the safety aspects relevant for rehabilitation robots can be more explicitly described in the Machinery Directive. Similarly to the applicability of the Machinery Directive, there might be standards from other domains which are more specific than the general safety and performance requirements listed in the MDR and can therefore be relevant for rehabilitation robots. As rehabilitation robots can have similarities with personal care robots as well as with collaborative robots, some of the relevant standards for these domains, like the ISO 13482 or ISO/TS15066, could be used for demonstrating specific essential health and safety aspects of the device related to the machine aspects of the device. Although robots as medical devices are out of scope for those standards, some methods as well as essential health and safety requirements might be relevant. However, the user has to consider any restrictions or differences between the domains and be aware that the respective standard is not directly applicable.

### State of the Art for Ensuring Safety of Collaborative Robots

Using the Machinery Directive (European Parliament and Council of the European Union, 2006) and the related harmonized standard ISO 12100:2010 *Safety of machinery—General principles for design—Risk assessment and risk reduction* (International Organization for Standardization, 2010), there is a typical workflow that any engineer focusing on safety of a collaborative robot system will follow. This workflow is described in (Saenz et al., 2020) together with useful examples, and includes the description of the system and the task (i.e., the robot, the environment, the users), the identification of hazards, an assessment of the resulting risk, and the identification of risk mitigation strategies. In addition to the documentation of the system and the risks involved, a validation of the risk mitigation strategies is also required. This validation is defined as a set of actions to evaluate with evidence that a set of safety functions meet a set of target conditions (Saenz et al., 2018), and is essentially a measurement to prove that a specific system complies with designated operating conditions characterized by a chosen level of risk. Currently there is no guidance from standards on how validation measurements should be executed.

The current cross-domain nature of robotics raises another dilemma for roboticists that many other users of the Machinery Directive and related harmonized standards do not encounter. This arises from the fact that the standards focusing on safety of collaborative robotics are domain-specific, i.e. for manufacturing or medical applications, and it is not always clear to a roboticist which standards are applicable to their system. Currently these standards covering different domains are not synchronized and can have conflicting requirements. This can lead to uncertainty, especially when robots are used in new domains (such as agriculture) or for multiple domains (i.e., an exoskeleton used for medical purposes or to support workers in manufacturing).

#### Concept Safety Skills

One proposed method to overcome these current challenges is based around the concept of safety skills. These have been proposed in (Saenz et al., 2018) and address the specific nature of human-robot collaboration by defining a set of abstract safety skills as the ability of a robot system to reduce risk. There can be different actual methods for implementation, depending on the specifics of the application, and these skills can be validated based on those application specifications at a system level.

The EU-funded project COVR (www.safearoundrobots.com) has developed this safety skills concept and the corresponding guidance for validation of these safety skills (in the form of so-called “protocols”) as a means for simplifying the process of ensuring safety for collaborative robots across all domains. While some safety skills would be familiar to engineers well-versed in the manufacturing domain, such as *maintain separation distance* (compare to Safety-rated Monitored Stop (SRMS) or Speed and Separation Monitoring (SSM) from the ISO/TS 15066) and *limit interaction energy* (compare to Power and Force Limiting (PFL) from the ISO/TS 15066), others might not be known within that domain. The safety skills were identified through a combination of a top-down and bottom-up approach, taken from safeguarding methods suggested in available robotics safety standards from different domains, as well as through an exhaustive analysis of hazards for known, possible robotics types for various domains.

The most important contribution of the concept of skills is that they offer engineers planning applications featuring collaborative robots a strong conceptual framework for considering risk mitigation strategies. Together with the associated protocols they offer clear guidance for how to execute the validation measurement, regardless of the domain. Since the skills and protocols reference and adhere to the currently available directives and safety standards, engineers are working within the current legal framework. The COVR project has developed a Toolkit (safearoundrobots.com) which users can use to identify relevant European Directives and Regulations, harmonized standards, and protocols based on their robotic application and safety skill used.

#### Concept Validation Protocols

As previously mentioned, the COVR protocols have been developed to support robotics application designers in the process of validating the completed systems. There are currently nineteen validation protocols available through the COVR Toolkit, with at least 12 more planned for the near future. These protocols are structured such that the required validation measurement (as part of the CE process) can be executed, and they are specific for valid combinations of robot devices and safety skills. The main sections of a protocol include:

• Introduction, including definitions and a specification of the scope and limitations of the protocol
• Description of the target behavior and metrics of the safety skill to be validated
• Description of the conditions (including the system description, eventual sub-systems, the environment, and other relevant aspects to consider for the validation measurement)
• Description of the measurement set-up including measurement devices, test arrangement, best practices for data acquisition
• Procedure (including eventual preparation, the test plan, test execution, data analysis practices, and suggestions for reporting and documentation)
• Eventual annexes with further information

These protocols can be considered to be an industry-wide best practice, providing guidance that goes beyond what is currently available in robotics safety standards on how to execute the validation measurement. They only look at system level behavior (not individual sensor functionality) and are closely related to the concept of safety skills. The protocols are testing procedures for safety validation and not to be confused with protocols used to evaluate safety during robot use, e.g., by monitoring adverse events in clinical trials (Nef et al., 2007; Borggraefe et al., 2010; Rodgers et al., 2019). While addressing comfort and safety during use of rehabilitation robots is important (Bessler et al., 2020), we are aiming to develop procedures for validating mechanical safety in the development phase, usually without a human in the loop. Ultimately, this aims to advance safety of a rehabilitation robot as much as possible before testing it clinically with (impaired) persons, potentially reducing the number or severity of adverse events occurring.

## Identified Hazards

Before safety skills and accompanying protocols can be applied to validate risk mitigation measures, risks need to be assessed based on the hazards associated with a cobot. Knowing which hazards need to be considered is therefore important to account for safety early in the design process of a rehabilitation robot and monitor it throughout its lifetime. This section provides an overview of frequent adverse effects of rehabilitation robot use and relates them to the underlying hazards.

**FIGURE 1:**
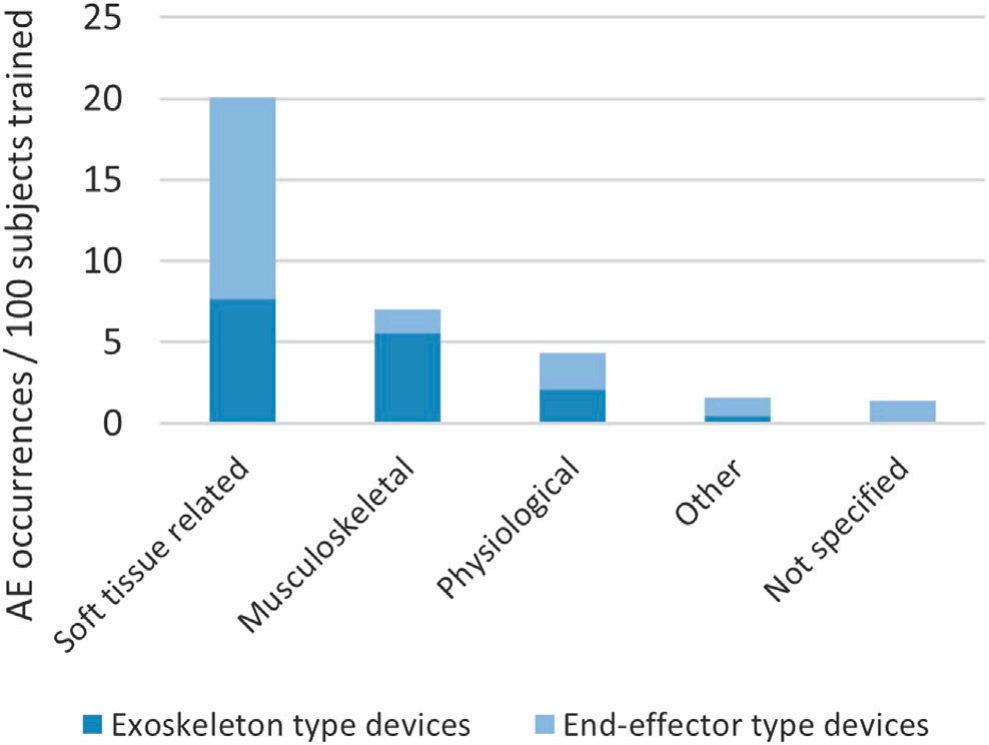
Types and occurrence of adverse events (AE) in stationary robotic gait training.

In a recent systematic literature review (Bessler et al., 2020), we collected information on occurrence and type of adverse events reported in connection with training in stationary robotic gait trainers. We counted approximately 17 adverse events per 100 subjects trained in a stationary robotic gait trainer. The adverse events were categorized with the most frequent types being soft tissue-related adverse events and musculoskeletal adverse events (Figure 1). The third category, physiological adverse events (e.g., sudden blood pressure changes), is regarded as unrelated to the mechanical setup of the robotic device in most cases, but to the being engaged in activity in general, and is therefore not analyzed in this article. Soft tissue-related adverse events in stationary gait trainers included skin irritation, skin reddening, skin abrasions, open skin lesions and bruising as well as discomfort and pain to soft tissue areas. Musculoskeletal adverse effects extracted from the systematic review were a tendinopathy, a tibia fracture, muscle pain, lower back pain, malleolus pain and discomfort and pain to joints.

In addition to general risk factors of the particular target population(s) for sustaining an injury, which include patient characteristics such as restricted blood flow, reduced sensation, uncontrolled muscle activities and low bone mineral density, there are risk factors which are connected to the design of the device. The soft tissue-related and musculoskeletal adverse events are all regarded to be attributable to forces exceeding safe limits.

In order to fulfill their function of supporting a certain movement in a patient, rehabilitation robots need to apply forces to the patient’s musculoskeletal system. Those forces are applied through contact points such as cuffs, straps, foot plates or harnesses and travel through soft tissues to the musculoskeletal system of the user. Excessively high forces can cause injuries both in soft tissue and in the musculoskeletal system. The nature and causes of those forces can however vary per event. As this principle of applying forces through contact points is the same for all rehabilitation robots, the following sections refer to not only stationary gait trainers, but rehabilitation robotics in general. Although the hazards identified through our recent review (Bessler et al., 2020) are based on incidents with stationary lower limb robots, comparable hazards have been reported in upper limb and mobile devices (Rocon et al., 2008; He et al., 2017; Schwartz et al., 2018; van Herpen et al., 2019). Although the interface mechanics between human and robot are often comparable, there are some obvious differences, such as lower weight bearing in upper limb rehabilitation robots and different location and surface area of device-skin interface. The device type and its design therefore have an influence on hazards to be considered. Nonetheless, the authors expect the identified categories of hazards to be relevant for all common rehabilitation robot types.

### Hazardous Forces at the Device-Skin Interface

Taking a closer look at hazardous forces that occur at the device-skin interface, we see that there is a need for further classification, based on the direction of the force in relation to the skin. Typically, there are normal forces and pressures, as well as shear forces and/or friction. In the following sections, these forces will be investigated in greater detail.

#### Normal Forces and Pressure

Normal forces and pressures are unavoidable, and even intended, at human-robot interfaces like the contact area between the patient’s skin and a cuff or harness. Circumferential pressures develop where a strap is tightened around a body part and local pressure areas are present where forces applied by the robot are transmitted through soft tissues and where gravity acts on the patient’s body weight that is supported by the robot. Too high pressures at the physical human-robot interface can obstruct blood flow or compress tissue, which can lead to injuries like bruises. Pressure injuries usually develop over bony prominences, where local pressure peaks arise (Sanders et al., 1995). Prolonged exposure to pressure, or pressure in combination with shear, can lead to pressure ulcers (National Pressure Ulcer Advisory Panel, European pressure ulcer advisory Panel, and Pan pacific pressure injury alliance, 2014; Hoogendoorn et al., 2017). Direction, distribution and duration of pressure are important factors for comfort and safety (Kermavnar et al., 2018b; Sposito et al., 2019b). Pressure magnitudes and distribution are influenced by the forces acting on the human-robot link, the surface area and shape of the interface, the compliance of the interface material and the characteristics of the body part to which the robot is attached (Sanders et al., 1995; Rocon et al., 2008; Varghese et al., 2018; Bader et al., 2019; Meyer et al., 2019). Moreover, external factors such as moisture, age, and preexisting conditions can have an effect on the soft tissue’s response to pressure (Sanders et al., 1995).

#### Shear Forces and Friction

Shear forces are forces in tangential directions which are oftentimes present at the interface between skin and robotic device in addition to pressures. Especially in dynamic situations, the robot’s movement is applying multidirectional forces to the human which are transmitted through the soft tissue. Moreover, shear forces and slipping at the connection points between human and robot can occur when the robot kinematics are different from the human kinematics (see section *Misalignment* below for a more detailed explanation) and the exoskeleton segments are therefore too long or too short in certain joint positions (Rocon et al., 2008; Akiyama et al., 2012). A part of this mismatch can be compensated by the compliance of soft tissue and cuff (Zanotto et al., 2015), but the shear forces, torques and slipping of cuff material on skin can nevertheless lead to soft tissue injuries or discomfort (Jarrassé and Morel, 2012; Akiyama et al., 2016).

The shear stress developing in soft tissues is influenced by the amount of pressure, the contact area between skin and attachment surface of the robot as well as by the friction coefficient that the robot surface material has with the human skin (Bergstrom et al., 1994). This interaction can be influenced by clothes worn underneath the robot cuff which add another layer of different friction coefficients between the cuff material and the skin. The coefficient of friction can in turn be influenced not only by the material but also by skin conditions such as humidity and surface topography (Motamen Salehi et al., 2018). Damp skin (i.e., small amount of water at interface) has a higher friction coefficient than dry skin, and wet skin (i.e., large amount of water at interface) has a lower friction coefficient than dry skin (Sanders et al., 1995). The material used at the human-robot interface therefore has an impact on the interaction between the robot attachment and the human soft tissue: While materials that have a low coefficient of friction with human skin might slip easily, materials with a higher coefficient of friction adhere to the skin and the shear acts in deeper layers of the soft tissue. Skin can react to shear and friction in various ways. When the friction is low and the movement repeated over a long period of time, the skin can adapt to the mechanical stress and get thicker by forming calluses (Naylor, 1955a; Sanders et al., 1995). Larger amounts of friction can lead to the formation of blisters where the friction force is transmitted through the surface layers of the skin (stratum corneum and stratum granulosum) and degenerates the deeper layer stratum spinosum. Clefts are produced which fill with fluid from the deeper dermis layer and the blister can rupture upon maintained mechanical stress leaving an open skin lesion (Naylor, 1955a). This type of skin response mostly occurs in areas with firm attachment of the skin to underlying tissues and a superficial layer thick and tough enough to form a roof on the blister. In areas with a thin superficial skin layer, shear forces and friction are more likely to cause an abrasion rather than a blister (Sanders et al., 1995). When skin slides over a rough contact material, chafing or abrasions of the surface layer(s) of the skin can occur.

Soft tissue injuries such as abrasions, skin lesions and discomfort to soft tissue are likely to be caused by interaction forces at the physical interface between human and robot. Due to the increased number and surface area of contact points in exoskeleton-type devices opposed to end-effector-type devices, one might expect that the risk of sustaining such an injury is higher when using exoskeleton-type devices. However, the above-mentioned systematic literature review (Bessler et al., 2020) showed that was not the case in stationary gait trainers (see also Figure 1). Many events of discomfort or injuries to soft tissue which were reported in end-effector-type device studies were attributed to the safety harness worn by the patient and the amount of body-weight support seemed to have an influence on the risk of soft tissue-related adverse events caused by the harness in exoskeleton-type devices but not in end-effector-type devices. All soft-tissue related adverse events related to straps or cuffs were however reported in exoskeleton-type devices.

### Hazardous Forces on the Musculoskeletal System

When the force exerted by the robot has traveled through the soft tissue and reaches the musculoskeletal system of the user, it can support or initiate movement of the human body. Too high forces can however cause harm to musculoskeletal structures such as ligaments, cartilage, muscles and bones. Not only the magnitude but also the direction and speed of applied forces play an important role in determining the injury risk.

#### Misalignment

When an exoskeleton is not perfectly aligned to the human skeleton, the exoskeleton joint axes will not be congruent with the human joint axes. Such resulting misalignments create undesired interaction forces which can reduce comfort and safety (Rocon et al., 2008). Misalignments can either be caused by a kinematic mismatch between the exoskeleton joint and the human joint or by poor fitting of the exoskeleton. An exoskeleton joint is always a simplified representation of the human joint which leads to unavoidable misalignments during movements. In addition to that, robotic devices for rehabilitation are usually one-size-fits-all solutions which, in contrast to customized medical devices such as orthoses and prostheses, can be worn by users of a range of body shapes and heights. Straps and segment lengths can be adjustable but the device is not custom-made for one patient and in rehabilitation settings usually used by several patients per day. Appropriate adjusting and fitting before each training session is therefore crucial to minimize the risk of injuries sustained due to misalignments. Besides causing excessive forces on the human musculoskeletal system (He et al., 2017), at the same time misalignment will cause high pressure and/or shear forces through slipping at the cuffs or straps (Rocon et al., 2008; Akiyama et al., 2012), see previous section. Forces that are not compensated for in the design of the robot or its interface will be transmitted to the musculoskeletal systems. If torques and forces are very high or acting in arbitrary directions on the musculoskeletal system, they can cause overloading and thereby pain and injuries to bones, joints and muscles (He et al., 2017; Mallat et al., 2019).

#### Exceeding Normal Range of Motion

Exceeding the physiological range of motion can lead to traumatic joint injuries such as ligament tears or capsule injuries (Hettinga, 1980; Verhagen et al., 2001). Besides those obvious and traumatic injuries, repeated overstretching and mechanical stress can lead to microscopic injuries which, when the mechanical stress remains, can in sum cause serious issues (Hettinga, 1980).

While misalignments only occur in exoskeleton-type devices, the risk of exceeding the normal range of motion is relatively easily avoidable in exoskeletons where the movements of the segments of the patients limb can be derived from and controlled by the movements of the exoskeleton segments. However, one has to consider that the range of motion of the user can vary according to patient-specific conditions or training aims and therefore has to be configurable to the patient. This can be achieved by adding an additional layer of safety features which is adaptable, for example using a software feature to reduce the range of motion. Further, force controls can be implemented which prevent for the human joints to be extended beyond safe limits. End-effector-type devices can only provide limited guidance for the movement to be executed. Arbitrary movements which apply excessive forces to the patient and lead to a joint exceeding the normal range of motion are a serious hazard associated with end-effector based rehabilitation robots (Rocon et al., 2008).

### Other Hazards Arising From Usage of Rehabilitation Robotics

Many of the injuries reported in literature that have been associated to the use of rehabilitation robotics can be attributed to the hazards above. However, additional events and failures can occur which can also lead to injuries sustained by rehabilitation robot users. For gait rehabilitation robots, falls present an important hazard. In stationary gait rehabilitation robots, body-weight support systems with a harness are often used to prevent falls. Falls of patients in lower limb exoskeletons or exosuits can be caused by a loss of balance, actuator failure or power failure (He et al., 2017). In contrast to healthy individuals, patients using lower limb exoskeletons already have a gait impairment and therefore have limited ability to recover from balance disturbances (van der Kooij et al., 2007; Haarman et al., 2017). Moreover, the robotic device disturbing the patient’s interaction with the ground and adding additional weight to the patient’s limbs are complicating factors. A common measure to avoid falls and maintain balance is the use of crutches. Features such as a “graceful collapse” can reduce the injury risk in the event of power failure. Some exoskeletons can detect falls and perform certain actions to reduce the injury risk (He et al., 2017).

In addition to the risks for the patient using a rehabilitation robot, one has to consider risks for bystanders and other types of users such as the physical therapist. The therapist is specially trained for working with the respective rehabilitation robot. Nevertheless, hazardous situations can occur when the therapist is supervising the training in close proximity to the robot. For example, a collision can occur where the therapist can sustain an injury due to an impact or due to being clamped between two robot segments or between a rigid object such as a wall and the robot. Wearable robots used for assistive purposes might be used in a home environment or other uncontrolled environments such as a park or shopping mall. In those situations, bystanders including children and pets are not trained in dealing with robotic devices and might behave in an unexpected way like moving into the robot’s trajectory or pushing a finger into an opening of the robot. Rehabilitation robot developers need to take those hazardous situations into account and avoid injuries e.g., by power and force limiting functions.

## Translating Hazards to Safety Skills and Validation Protocols

The hazards detailed in the previous section have proven to be relevant in rehabilitation robotics based on reports of adverse events in literature. To achieve the goal of a safe physical interaction between rehabilitation robots and their users, the risks connected to those hazards need to be addressed in the safety validation process. The authors propose to evaluate the mechanical safety of rehabilitation robots by validating safety skills (see section *Concept safety skills*). This way, the same approach can be used independent of how the safety function is technically implemented as long as the same safety skill is addressed.

As a first step, safety skills were identified based on the hazards described in previous sections (Table 1). By analyzing the hazards extracted from the reported adverse events, and methods to mitigate the related risks, the most relevant safety skills for rehabilitation robots were identified and linked to the corresponding hazard(s). Subsequently, for each of the identified safety skills, testing protocols are defined that describe how a particular safety skill should be assessed. In the following sections, each safety skill is described in more detail and the basic concepts for validating those safety skills are explained (detailed validation protocols can be found on the publicly available online COVR Toolkit which is currently under development (safearoundrobots.com)). In the future, additional safety skills relevant for rehabilitation robots might be identified and new protocols developed. We highly recommend performing all safety tests with dummies and simulators instead of a human to avoid dangerous situations during safety testing (COVR, 2019).

**TABLE 1:** Overview of identified hazards, influencing factors, potential injuries and related safety skills. Note that this list is not extensive and additional relevant hazards and safety skills might be identified in the future. (Icons © COVR).

Hazard	Influencing factors	Device part (device type)	Potential injuries	Safety skill
Continuous or repetitive pressure exceeding safe limits	- Design and fit of mechanical interface (pressure distribution/peaks, high circumferential pressure)	- Cuffs/straps (mostly exoskeleton devices) - Harness (stationary devices)	Soft tissue-related (e.g., bruises, pressure ulcers)	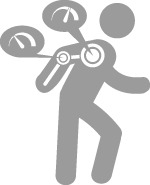 Limit restraining energy
Shear forces exceeding safe limits	- Direction, speed, pressure, duration - Material at human-robot interface (friction, microclimate) - Sliding of mechanical human-robot interfaces (improper fit, misalignment)	- Cuffs/straps (mostly exoskeleton devices) - Harness (stationary devices)	Soft tissue-related (e.g., skin abrasions, blisters, skin lesions)	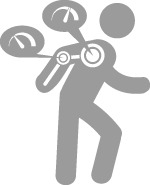 Limit restraining energy
Misalignment	- Direction and amount of misalignment (translational vs. rotational misalignment, micro vs. macro misalignment)	- Exoskeleton joints (only exoskeleton devices)	Musculoskeletal (e.g., joint pain/injuries, bone fractures);Soft tissue-related (e.g., skin abrasions, bruises)	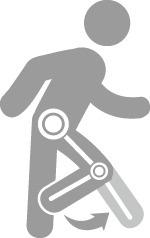 Maintain proper alignment
Exceeding physiological range of motion	- Direction, force, speed	- Exoskeleton joints or end-effectors (mostly end-effector devices)	Musculoskeletal (e.g., joint pain/injuries, muscle strain)	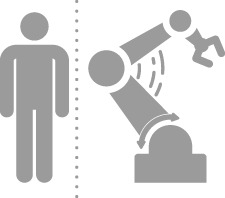 Limit range of movement
Collision with bystander	- Impact/clamping force, speed, weight - Environment (walls that can cause clamping), surface material and shape	- Moving parts (all device types)	Various (e.g., bruises)	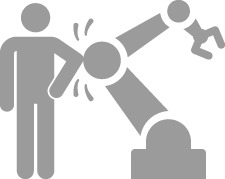 Limit physical interaction energy

### Limit Restraining Energy

Many rehabilitation robots are strapped to the patient during use, which means that they exert a restraining energy on the user, for example at the site of a cuff (see also the definition of restraint type physical assistant robot, ISO 13482 (International Organization for Standardization, 2014)). In order to ensure a safe interaction between the restraining parts of the robot and the human skin and soft tissue, the safety skill *limit restraining energy* needs to be validated. The safety tests will need to assess whether the interaction forces at the physical interfaces between human and robot stay within safe limit values. The forces present are repetitive and/or continuous forces and are exerted in different directions, which can have various negative effects on the skin and soft tissue of the user as explained above. It is expected that the stress concentration at the edge of a robot cuff is highest (Giannakopoulos et al., 2001) which is why special attention should be given to the forces present at those locations. Moreover, the presence of pressure peaks over bony prominences is a known phenomenon (Bader et al., 2019). Therefore, it is advised to assess the distribution of interaction forces over the whole interface or at least at the locations with the expected highest stress concentrations.

Pressures and their distributions can be assessed with thin and preferably flexible sensors mounted to the robot’s connection surface or to a dummy limb’s artificial skin. During testing, the robot is performing movements which lead to force application to the dummy at the site of the robot’s restraining part representative for normal use. In some cases, it might not be possible to perform the test with the complete robot setup as it is used in practice. Wearable (i.e., non-stationary) exoskeletons might have to be fixed to a rigid frame or the execution of the forces might have to be performed by a separate robotic manipulator equipped with the cuff to be tested and programmed to execute the same forces as the rehabilitation robot would. Where there are different training situations with varying pressure levels, the worst-case scenario as identified in the risk management process is used for testing. The robot passes the safety skill validation test if the pressures remain within the safe levels at all times.

The concept of performing a safety validation test for shear forces is similar. Thin and flexible shear sensors are used instead of pressure sensors. Alternatively, a piece of porcine skin can be attached to the dummy surface and analyzed for any damages after applying the shear force that is applied by the rehabilitation robot surface during normal use. When there is no damage to the porcine skin and no recorded shear forces higher than the defined safe limit values, the validation test is passed.

### Maintain Proper Alignment

Strategies to avoid negative effects of misalignment to the user’s musculoskeletal system must be considered, especially in exoskeletons. The validation of the corresponding safety skill *maintain proper alignment* includes measuring the forces applied to a user’s joint and checking whether they exceed a safe limit value or not. As mentioned above, those validation tests cannot be performed on a human user for safety reasons. Therefore, a dummy limb is needed to enable measuring of internal joint forces. The instrumented dummy used for this validation test consists of two rigid tubes connected by a joint mimicking the human bones and joint, and a compliant material mimicking the soft tissue. One of the tubes is equipped with a six degrees of freedom force and torque sensor. During the experiment, the instrumented dummy limb is fastened to the exoskeleton to represent the user’s limb in a normal use situation. The exoskeleton joint is then moved through its usual range of motion with different misalignment settings. A misalignment can either be a translation of the exoskeleton flexion axis relative to the flexion axis of the human (dummy) joint or a rotation of the robotic flexion axis relative to the joint flexion axis of the human. The exoskeleton passes the validation test if the maximum torques and forces acting on the dummy joint at no point in time exceeded the safe limit values.

As explained in previous sections, a misalignment can not only lead to injuries to the musculoskeletal system but can also lead to soft tissue injuries as the cuffs or straps might shift or exert a higher pressure than during well-aligned situations. It is therefore advised to perform the validation protocols of the safety skill *limit restraining energy* under the condition of different misalignments to test for the effects of misalignments on the human-robot interaction on soft tissue level.

### Limit Range of Movement

The range of movement for a rehabilitation robot has to be restricted in such a way that the physiological range of motion of the patient inside the robot is not exceeded. The safety skill *limit range of movement* can be relevant for a number of device types where the robot initiates or supports movement of limbs. For the validation of single degree of freedom joint range of movement (usually in exoskeletons), a simple test setup using an angle measurement device such as an electro-goniometer can be used. A dummy payload is attached to the exoskeleton which is then programmed to move back and forth over the specified angular range of motion at maximum angular velocity. The exoskeleton joint angles are measured over time and compared with the safe range of movement. If the safe range of movement is exceeded at no point in time, the validation test is passed.

For more complex joints and end-effector devices, the spatial range of movement can be assessed using a marker-based motion analysis system. Markers are attached to a reference point, based on which the safe spatial range of movement is defined, and to an endpoint that relates to the simulated human body part attached to the robot. The robot is then programmed to move the endpoint through the safe range of motion, along the borders of the safe range of motion and also to coordinates outside of the safe range of motion that are within the robot’s reach. The robot passes the validation test if the endpoint exceeds the safe range of motion at no point in time.

### Limit Physical Interaction Energy

To account for the safety of therapists working with a rehabilitation robot as well as bystanders, the hazard of accidental contacts such as collisions with a rehabilitation robot or a part thereof has to be considered. The skill *limit physical interaction energy* protects bystanders from injuries caused by collision with a rehabilitation robot. The concept of the validation process of this safety skill is to simulate an accidental contact of the rehabilitation robot with the human by evoking a contact of the robot with a bio-fidelic measurement instrument that mimics the biomechanical characteristics of the human body. The bio-fidelic measurement instrument consists of a load cell connected to an impactor via an interchangeable spring (Huelke and Ottersbach, 2012). On the other side, the load cell is connected to a rigid housing and the impactor is covered by a soft damping material. The combination of the spring and the damping material are chosen in such a way that it has the same biomechanical characteristics as the human body or the body part with which the collision can occur. A foil sensor for pressure measurement is mounted on top of the measurement instrument. To execute the validation test, the measurement instrument is fixed in a position in which the accidental contact with the robot can occur. The robot or exoskeleton is then instructed to move along a set trajectory with a pre-defined speed reflecting normal use or the worst-case situation. The impact forces and pressures are analyzed based on safe limit values for transient contacts in which the human segment that gets into contact with the robot can move freely and/or clamping contacts in which the robot clamps the human against a rigid object such as a wall or the rigid support structure of a robot. If the safe limit values for forces and pressures are not exceeded in any of the test repetitions and conditions, the validation of the safety skill is passed.

## Discussion

By combining existing knowledge about reported injuries of gait robots with potential underlying mechanisms in relation to physical human-robot interaction, we have identified five main hazards relevant to physical safety of humans interacting with rehabilitation robots. These five hazards were related to four different safety skills. These were used as a framework for describing safety validation protocols for each of those skills that are broad enough to be used for a range of rehabilitation robot types. Some of those protocols are publicly available in detail, in draft version or currently under development. Nevertheless, this analysis also highlighted particular knowledge gaps that are yet to be closed to achieve a sufficient set of clear guidelines for rehabilitation robot developers. These gaps and associated needs for simplifying safety validation of rehabilitation robots are discussed below.

### The Potential and Limitations of Cross-Fertilization Among Domains

Since rehabilitation robotics is a relatively young and highly dynamic field, availability of specific standards, validated testing procedures and safe limit values is limited. It can therefore be beneficial to consult standards or best practices from other domains that deal with similar safety issues. For example, testing procedures are more specific in standards and technical reports or technical specifications for collaborative robots in industry, some of which are also relevant to rehabilitation robots. For the safety skill *limit interaction energy*, best practices and methodologies from ISO/TS 15066 (International Organization for Standardization, 2016) can be adapted for use with rehabilitation robotics. Further, even though EN-ISO 13482 and the corresponding test methods (International Organization for Standardization, 2014; International Organization for Standardization, 2020b) are limited to personal care robots, excluding medical applications per definition, some of the methods can be taken into consideration when developing safety validation methods for rehabilitation robotics. However, the applicability of non-medical standards to medical devices is limited. One has to consider differences in use and most importantly the vulnerability of patients using rehabilitation robots. Therefore, one cannot use thresholds or limit values stated in non-medical standards. Moreover, the cognitive and/or physical abilities of rehabilitation robot users are most likely restricted compared to factory workers which has to be taken into consideration during risk assessment.

### Gaps and Needs in Research for Producing and Executing Protocols

#### Measurement Techniques and Devices

There is a pressing need for reliable and accessible measurement methods to assess the physical interaction between humans and rehabilitation robots. Regarding the assessment of normal forces present at the interface between human skin and robot contact surface, a means to measure pressures or normal forces and their distribution over the contact area would be most beneficial. Pressures and shear forces at the interface between cuffs and human soft tissue are distributed unequally with peaks at the sites of bony prominences and along the edges of the cuff (Sanders et al., 1995; Giannakopoulos et al., 2001; Bader et al., 2019). Load cells measuring the net force exchange between the human and robot are therefore deemed insufficient to assess forces that might harm the rehabilitation robot user on soft tissue level.

Pressure mats have been used for analyzing contact surfaces in e.g., backpacks (Wettenschwiler et al., 2017), wheelchairs (Apatsidis et al., 2002; Tam et al., 2003; Pipkin, 2008), beds (Defloor, 2000; Moysidis et al., 2011; Hemmes et al., 2017) and prosthetics (Rajtukova et al., 2014; Al-Fakih et al., 2016), and also in previous experiments to measure interface pressures in exoskeletons (Levesque et al., 2017; Huysamen et al., 2018) or soft exosuits (Xiloyannis et al., 2019). For this purpose, pressure mats must be flexible and compliant as the contact surface of a rehabilitation robot is usually curved and a stiff sensor would change the physical human-robot interaction. While those pressure mats are available in a variety of sizes, pressure ranges and sensitivities, they are not easily adjustable for use in many differently shaped devices. One needs a pressure mat that fits in the respective cuff or other contact surface without overlapping and that has an appropriate measurement range and sensitivity for the contact situation under consideration. Weight bearing interfaces such as footplates or harness straps will yield much higher contact pressures than for example a forearm splint.

An affordable alternative for commercial pressure mats are thin film polymer sensors called Force Sensitive Resistors (FSRs). FSRs are often used in commercial pressure mats, where they are arranged in a matrix, but can also be purchased as single sensors which can be arranged in groups or used individually. Advantages of FSRs are their thinness, low cost, sensitivity and sensing range. They are commercially available in different shapes and sizes and can be assembled in grids, which makes them suitable for many biomechanical applications including interface pressure measurements in rehabilitation robotics (Tamez-Duque et al., 2015; Rathore et al., 2016; Levesque et al., 2017). However, FSRs also have limitations, including sensor drift and hysteresis (Dabling et al., 2012). In addition to that, surface characteristics such as curvature and compliance as well as temperature can have an impact on the measurement outcomes (Schofield et al., 2016). When single FSRs are used at a human-robot interface, a portion of the applied force is getting lost as it is distributed over not only the sensing area of the FSRs but also the edges of the sensors and the space in between FSRs (Bessler et al., 2019). A method to avoid this problem is to glue “pucks” or semi-spheres to the sensing area of the FSRs to apply the force to that area only (Castellini and Ravindra, 2014; Levesque et al., 2017). However, these added structures will change the pressure distribution by introducing an additional, uneven layer to the human-robot interface. Recent experiments have shown that the thin FSRs left indentations on the skin, leading to the assumption that an added layer of “pucks” or semi-spheres will introduce pressure peaks at the edges of those structures (Bessler et al., 2019).

The continuous or repetitive shear force present at the interface between a rehabilitation robot and the user’s soft tissue is expected to have a larger effect on the soft tissue injury risk than the pressure (de Wert et al., 2015; Klaassen et al., 2019). It is therefore of utmost importance to have reliable measurement methods available. A lot of research has gone into the development of thin sensors for interface stress measurements, especially in prosthetic sockets and shoes (Al-Fakih et al., 2016; Lazzarini et al., 2019). A concept that has received much attention and seems promising is a sensor that works based on capacitance changes due to deformation of a polymer pillar structure (Lee et al., 2008; Cheng et al., 2010; Laszczak et al., 2015; Liang et al., 2015). Those thin and flexible sensors have been used for different application areas, including prosthetic socket interfaces and fingertip contact forces (Laszczak et al., 2016; Valero et al., 2016; Tang et al., 2017), and might also be suitable for assessing the interface pressure and shear at contact areas between rehabilitation robots and their users. Georgarakis et al. (2018) suggested a setup where a force sensor with three degrees of freedom is built into a cuff. As opposed to the reliability and robustness of load cells, capacitance sensors can be more prone to errors. A general drawback of using sensors for assessing the effects of shear forces is that the sensor material adds another layer to the interface between human and robot which, based on the changes in surface topography and friction, can alter the physical human-robot interaction.

A different approach for assessing safety of shear forces applied to the human soft tissue is to apply the same forces to a surrogate skin and analyze it for any damage. Porcine skin is widely accepted as surrogate for human skin as the macroscopic morphology, cutaneous blood supply and wound healing characteristics are similar (Schwartz et al., 2018). The proposed method to test restrained-type physical assistance robots is to fix a piece of porcine skin to a dummy of a shape and compliance comparable to the human body part (Aso et al., 2013) and apply the forces that would be applied by the rehabilitation robot during normal use (Akiyama et al., 2015; Akiyama et al., 2016) using a manipulator. After that, the porcine skin sample is analyzed under the microscope to check for signs of skin damage (Mao et al., 2017b; International Organization for Standardization, 2020b). This method is a way to validate the skill *limit restraining energy* by showing that normal use of the rehabilitation robot does not lead to skin injuries. It does however have some limitations as it requires the use of porcine skin specimens and the availability of special equipment such as a cryostat and microscope. Moreover, porcine skin does have a morphology comparable to the one of human skin but it is considerably thicker (Schwartz et al., 2018) and might therefore differ in its reaction to physical stress. To avoid using porcine skin for safety testing, Mao et al. (2017a) have developed an artificial dummy skin for abrasion tests. However, its comparability in abrasion damage onset with human skin has not yet been validated.

Measuring the effect of rehabilitation robot use on internal body structures remains another challenge. In addition to ethical considerations about safety testing with humans, it is also technically impossible to directly measure the stress on bones and joints *in vivo*. Instrumented dummies therefore appear to be the only reasonable option for assessing the safety skill *maintain proper alignment* and other safety skills for limiting the forces applied to the musculoskeletal system. While crash test dummies are a gold standard for assessing the impact of collisions on the human body in the car industry, those dummies are very costly and not optimized for assessing physical human-robot interaction in continuous contacts. Akiyama et al. (2012) proposed a dummy leg setup to measure the effects of misalignment at the cuff locations. However, the skeletal system will be reached by a part of the applied force only as the soft tissue has a dampening function. It might therefore be more beneficial to use a setup with force and torque sensors included in the ‘skeletal’ structure of the dummy limb to investigate the effects of misalignment on the skeletal system. Complex systems are needed to replicate the physical interaction between humans and robots. A simplification of joints used for dummy limbs can resolve the kinematic mismatch that would be present between a human joint and the simplified rehabilitation robot joint. Therefore, complex, potentially actuated joints that can replicate the rehabilitation robot user’s behavior are needed. There is no validated measurement system or best practice available yet for developers to use for safety testing of their device.

#### Safe Limit Values

To perform safety validation of a rehabilitation robot, the developer not only needs information on how to test the respective safety skill, but also on the safe limit values that can be used as a pass/fail criterium for the test. While much research has been performed on safe limit values for accidental contacts such as collisions between a factory worker and a collaborative robot, little is known about acceptable force magnitudes for continuous contacts with patients.

Pain onset thresholds have been investigated for pressures and forces applied during accidental contacts such as collisions or clamping situations with a collaborative robot (Behrens and Elkmann, 2014; Behrens and Pliske, 2019; Melia et al., 2019). Those limit values for many different locations on the human body have been adopted for ISO/TS 15066 (International Organization for Standardization, 2016). They might also serve as guideline for accidental contacts between rehabilitation robots and bystanders. However, one has to keep in mind that the underlying pain onset experiments were performed with healthy adults. Therefore, while the biomechanical limit values might be applicable for physical therapists, they are not for certain other types of bystanders such as children, elderly persons or patients. In addition, contacts in collaborative robots are typically the result of foreseeable misuse or technical failure (Behrens and Pliske, 2019), while they are intended in rehabilitation robots. Therefore, those limit values are explicitly applicable to accidental contacts only (i.e., safety skill *limit interaction energy*), which means that they cannot be used for those continuous contacts usually present between a rehabilitation robot and patient (i.e., safety skill *limit restraining energy*). In fact, it has been reported that after two or three repeated measurements at the same location, effects on the skin such as bruises, reddening and skin abrasions were observed (Muttray et al., 2014). Moreover, sustained pressure at a level of half the magnitude of the pain onset threshold becomes painful after a few minutes (Belda-Lois et al., 2008). In rehabilitation robot use, the human body is exposed to sustained pressure, often including cyclic loading and unloading phases. Such intermittent pressure at low frequencies can be perceived as very uncomfortable as it leads to summation of pain (Kermavnar et al., 2018b; Sposito et al., 2019b).

Depending on the rehabilitation robot type, the pressure can for example be exerted through cuffs, harnesses, straps or splints. Comfort and pain onset thresholds should therefore not be assessed with indenters which have a relatively small contact surface but with algometers that mimic the interface of the rehabilitation robot. Previous research has therefore focused on circumferential tissue compression. Acceptable levels of circumferential pressures are expected to be about 20 times lower than single point pressure pain onset thresholds such as the ones indicated in ISO/TS 15066 (Kermavnar et al., 2018b). More specifically, a systematic review found pain detection thresholds in healthy persons (i.e., pain onset thresholds; perceived discomfort) for circumferential pressure of between 16 and 34 kPa and pain tolerance thresholds (i.e., the pain becomes unbearable) of 42–91 kPa (Kermavnar et al., 2018b). Another systematic review states that circumferential pressure limit values for chronic pain patients are significantly lower, with pain onset thresholds of about 10–18 kPa and pain tolerance thresholds of below 25 kPa (Kermavnar et al., 2018a).

Further, the increase of pain with time was higher and the adaptation to pain lower in patients with chronic pain compared to healthy subjects. Recent studies have investigated the relationship between perceived comfort and circumferential pressure applied using pneumatic cuffs of different widths to participants’ thighs and shanks during standing and walking (Kermavnar et al., 2020a; Kermavnar et al., 2020b). They discovered that the discomfort and pain thresholds were lower during walking than during standing still and that narrower cuffs as well as anatomical sites with smaller volumes of soft tissue (shank as compared to thigh) lead to higher thresholds. These findings are bringing us an important step closer to comprehensive guidelines for safe limit values for pressure in rehabilitation robot use. However, the pressures were only applied for a relatively short duration of 60 s, which does not represent normal use of most rehabilitation robots. Therefore, more research is needed to identify acceptable limit values of sustained and cyclic pressure including the relationship between pressure magnitude and exposure time.

An additional challenge with regard to safe limit values for pressures are the varying patient characteristics. Most studies investigating pain pressure thresholds have been performed with healthy individuals. However, the systematic review mentioned above (Kermavnar et al., 2018a) identified that pain perception differs significantly between healthy individuals and patients with chronic pain. Moreover, the reaction to contact pressure is highly dependent on factors such as the anatomical structure, tissue composition and stiffness, blood flow, lymphatic flow as well as the individual health status and diseases affecting inflammation and repair capacities (Bader et al., 2019). As all these factors can be influenced by the condition of the common rehabilitation robot user, it remains very challenging to define clear thresholds.

Regarding acceptable limit values for shear forces applied by rehabilitation robots, the lack of reliable methods for assessing interface shear as explained above is a major limiting factor for research on comfortable and safe levels of shear forces at the interface between human soft tissue and robot contact surfaces. Based on research on the development of friction blisters with human participants (Naylor, 1955b; Naylor, 1955a) as well as porcine skin (Mao et al., 2017b), a relationship of tangential traction and time resulting in a threshold curve of inherently safe shear force (reaching from about 40–45 kPa at 3 min to about 25–30 kPa at 23 min) has been identified. As these experiments have been performed with a stainless steel plate which clearly has different characteristics than a cuff used in rehabilitation or physical assistant robots, the results were validated using a cuff mounted on a manipulator (Mao et al., 2017b). The results have been adopted in ISO/TR 23482-1 (International Organization for Standardization, 2020b). However, one has to consider that these limit values have been obtained from experiments with porcine skin and the reaction of human skin might be different. The reaction of human skin to friction has been investigated in the past (Naylor, 1955a), but the forces were only applied for a few minutes which does not represent normal rehabilitation robot use. The validation experiment using a cuff was based on net tangential forces applied by the rehabilitation robot over the whole cuff. Therefore, there is no information on the local magnitudes of applied shear which can be influenced by the cuff material and skin humidity and might not be evenly distributed over the contact surface of skin and cuff.

Furthermore, the limit values have been developed for exposure times of up to 23 min and rehabilitation robots might be used for durations longer than that. It is important to realize that the levels acceptable for shear stresses in rehabilitation robots should be clearly lower than the available limit values as development of friction blisters during rehabilitation robot use is inacceptable and depending on the patient’s health status, blisters can lead to complications. Moreover, it has been shown that stresses on the skin increase with an increasing coefficient of friction present at the interface. The friction coefficient of skin and cuff depends, among other factors, on the curvature, the material used and the humidity at the interface (Derler and Gerhardt, 2012; Behrens and Elkmann, 2014; Bader et al., 2019). The individual mechanisms behind a soft tissue injury can be difficult to identify as many factors can play a role. While the microclimate and skin condition clearly have an influence on susceptibility for skin injuries, the influence of hair is unclear (Derler and Gerhardt, 2012). More research is therefore needed on the influence of different environmental conditions and cuff materials on injury mechanisms. In addition to that, the increase in shear stress with an increasing coefficient of friction is higher in stiffer skin tissue, which occurs with aging and in certain conditions such as diabetes (Shaked and Gefen, 2013). This is an indication that the same applies regarding the influence of patient characteristics on the injury risk, as for pressure limit values. These factors present a major challenge, and a pressing need, for defining clear limit values applicable for different patient groups and under normal use conditions of rehabilitation robots.

Normal physiological ranges of motion have been documented in literature and are common knowledge in clinical practice (Soucie et al., 2011). However, rehabilitation robot developers have to consider that their target group might have limitations regarding their passive range of motion, for instance due to contractures. For rehabilitation robots used in a clinical setting, the range of motion could be individually set by the patient’s therapist.

Regarding forces and torques applied to musculoskeletal structures, it is difficult to define acceptable limit values. They could be based on voluntary joint torques that can be applied by healthy individuals (Anderson et al., 2007; Hahn et al., 2011) or on torques applied by therapists during conventional gait training (Galvez et al., 2005; Veneman et al., 2007). Moreover, knowledge on bone fracture occurrence in accidents can be taken into consideration (Soni et al., 2007; Takahashi et al., 2012; Fujikawa et al., 2017; International Organization for Standardization, 2020b), although it has to be considered that rehabilitation robot users are exposed to continuous and cyclic forces which is a situation very different from impacts during accidents. Moreover, similar to all other categories above, the tolerance for forces and torques applied by a rehabilitation robot, for example due to misalignments, can be lower for patients than for healthy individuals. One reason for that can be a reduced bone mineral density which is common, for example, in spinal cord injury patients and can lead to an increased risk for bone fractures (Eser et al., 2005).

#### Safety by Design

Through inherent safety, or safety by design, hazards can be addressed and minimized early in the design process. Safety features therefore should not only be added to existing devices, but the relevant hazards should already be mitigated by design features. To enable this, more research is needed on best suited materials and technologies. For example, the knowledge about optimal material choice for the physical human-robot interface is limited. Even in more established domains like orthotics and prosthetics, soft tissue injuries are a recurring problem and there is no gold standard or perfect solution (Bader et al., 2019).

To mitigate the hazard of misalignments by design, much research went into compensation mechanisms such as passive joints (Rocon et al., 2008; Stienen et al., 2009). These can work well for stationary devices, but make the device heavier and bulkier, which makes them unsuitable for truly wearable exoskeletons (Rocon et al., 2008), unless the robot inertia is actively compensated (Zanotto et al., 2015). Research suggests that misalignment in lower limb exoskeletons lead to increased forces mainly at the thigh cuff which could be compensated by compliant cuffs (Zanotto et al., 2015), however, the suitability and effectiveness of such a compensation strategy would have to be validated.

## Conclusion

In the present review on safety assessment of rehabilitation robots, we pointed out the state of the art and needs regarding guidelines for evaluating rehabilitation robot safety, to provide directions for developers to design safe rehabilitation robots. Rehabilitation robotics is a very diverse and dynamic field which contributes to the complexity of its safety validation. In addition to that, the nature of physical contacts between rehabilitation robots and patients, which introduce intended continuous and cyclic interaction forces, poses a challenge. There is a lack of clear recommendations for safety testing in rehabilitation robot specific legislation and standardization. While the transition from MDD to MDR increases the focus on safety, developers and manufacturers need more precise and practical guidelines. The experience with collaborative robots in domains like manufacturing can potentially help to reach this goal as there are more best practices available than in the healthcare domain. The concept of safety skills described in this paper makes use of cross-fertilization by defining domain-unspecific abilities of collaborative robots to reduce a risk, which can be validated according to structured validation protocols (www.safearoundrobots.com). Those protocols reflect industry-wide best practices and can be considered as guidelines for safety validation of collaborative and rehabilitation robots on a system level.

Based on knowledge about adverse events occurring in rehabilitation robot use, we identified excessive forces on the soft tissue level and on the musculoskeletal level in different directions as most relevant hazards for rehabilitation robots and related them to four safety skills, providing a concrete starting point for safety assessment of rehabilitation robots. We further identified a number of gaps and research needs which need to be addressed in the future to pave the way for more comprehensive guidelines for rehabilitation robot safety assessments. Predominantly, besides new developments of safety by design features, there is a strong need for reliable measurement methods as well as acceptable limit values for human-robot interaction forces both on skin and joint level.
